# Surgical management of metastatic cutaneous Crohn’s disease: a case series from a tertiary centre in the United Kingdom

**DOI:** 10.1007/s10151-026-03313-9

**Published:** 2026-05-29

**Authors:** D. Selvakumar, D. Leiberman, A. O’Connor, C. C. Lyon, A. Brass, J. McLaughlin, R. Winterton, S. M. Cruickshank, L. Hancock

**Affiliations:** 1 Lydia Becker Institute of Immunology and Inflammation, University of Manchester, AV Hill Building (75), Upper Brook Street, Manchester, M13 9PT UK; 2 Manchester University Hospitals NHS Foundation Trust, Manchester, UK; 3 Department of Dermatology, York Hospital, York, UK; 4 Department of Dermatology, Salford Royal Hospital, Northern Care Alliance NHS Foundation Trust, Salford, UK; 5 Manchester Academic Health Science Centre, University of Manchester, Manchester, UK; 6 Department of Gastroenterology, Salford Royal Hospital, Northern Care Alliance NHS Foundation Trust, Salford, UK

**Keywords:** Crohn’s, IBD, Proctectomy, Cutaneous, Metastatic

## Abstract

**Background and aims:**

Metastatic Crohn’s disease (MCD) is a rare extra-intestinal manifestation (EIM) of Crohn’s disease (CD), defined by granulomatous inflammation of skin non-contiguous to the gastrointestinal tract. This study describes the clinical features, surgical management and wound healing outcomes of the largest national surgical cohort of patients with severe MCD, a topic that remains poorly represented in the literature.

**Methods:**

This retrospective single-centre case series included adults (> 18 years) undergoing surgical management for MCD from 2019 to 2024. Diagnosis was confirmed by expert clinical and histopathological assessment. We describe pre-operative optimisation, individualised surgical approaches and post-operative management delivered by a multi-disciplinary team led by consultant colorectal and plastic surgeons. Wound healing was assessed clinically in a specialist complex wounds clinic at 6 and 12 months and at final long-term follow-up.

**Results:**

Eleven female patients (median age 37 years) underwent surgical intervention. At 6 and 12 months, 45.5% achieved complete healing, improving to 81.8% by final follow-up (median 36 months). Most underwent a combined medical and surgical approach with proctocolectomy and tailored perineal reconstruction. Persistent lesions and non-healing ulcers required additional adjuncts to treatment which included topical tacrolimus, hyperbaric oxygen therapy and surgical re-excision to improve healing outcomes.

**Conclusions:**

MCD is a challenging condition requiring surgical treatment in refractory cases despite medical therapy. Our study highlights the need for multidisciplinary working alongside meticulous pre-operative optimisation and close post-operative follow-up to improve long-term wound healing outcomes for patients with this rare and complex disease.

## Introduction

Metastatic Crohn’s disease (MCD) is an uncommon but debilitating extra-intestinal manifestation (EIM) of Crohn’s disease (CD) first described by Parks et al. in 1965 [1]. It is characterised by non-caseating granulomatous inflammation of the skin at sites anatomically separate from the gastrointestinal tract. Importantly, MCD represents a distinct cutaneous manifestation of CD, which may occur alongside perianal Crohn’s disease (pCD) or independently, rather than representing a direct extension or progression of perianal disease. Lesions most commonly affect intertriginous, perineal and genital skin, but may occur at any cutaneous site. People can present with MCD symptoms before, concurrently or years after symptoms of luminal CD, compounding the diagnostic challenge [2]. Its heterogeneous clinical presentation often results in patients being assessed across multiple specialties, including primary care, gastroenterology, colorectal surgery, gynaecology, urology and dermatology. In the absence of coordinated multidisciplinary care, delays in diagnosis and initiation of appropriate treatment are common. Despite increasing recognition, the underlying pathogenesis of MCD remains poorly understood [3].

There are currently no established diagnostic or treatment guidelines for MCD. Several treatment modalities have previously been reported, in case reports and series. Treatment options include topical and systemic therapies such as antibiotics, corticosteroids, thiopurines and biologic therapies. Surgery is an important treatment option for lesions refractory to medical therapy. However, data describing and assessing surgical management are limited, with only one previously reported case series which predated the biologic era [4]. This case series explores the clinical features, surgical management and outcomes of patients with MCD, providing insight into preoperative optimization, surgical decision-making and postoperative care for patients with this rare and complex disease.

## Methods

### Study design

This is a retrospective single-centre consecutive case series of adult patients undergoing surgical intervention for the management of MCD between January 2019 and January 2024 at the Manchester University NHS Foundation Trust, a large tertiary academic institution in Manchester, UK.

All research was conducted in accordance with the Declaration of Helsinki principles. This study (IRAS ID: 321786) was reviewed and approved by the Wales Research Ethics Committee (REC reference: 23/WA/0164). All identifiable patient information was removed, and all analyses were performed using anonymised data.

### Participants

Adult patients (> 18 years age) were identified through review of inflammatory bowel disease (IBD) multi-disciplinary meeting (MDT) lists, gastroenterology and colorectal surgery clinic lists. Those without a definitive clinical or histological diagnosis of CD were excluded.

MCD was diagnosed by combined expert clinical assessment and histological diagnosis on skin biopsy, which was the presence of granulomatous inflammation in the dermis or epidermis of the skin. Additional stains for acid-fast *Bacilli* were performed to exclude other causes of granulomatous inflammation such as tuberculosis. Hidradenitis suppurativa (HS) was excluded on the basis of clinical and histopathological examination. Patients were also screened against guidelines for monogenic Crohn’s testing and excluded if they met criteria for genetic testing [5]. Patients underwent careful and thorough counselling with consultant colorectal and plastic surgeons prior to undergoing surgery.

### Pre-intervention patient optimisation

Multi-modal pre-operative optimisation was an essential part of treatment. Expert assessment by specialist dietitians and, if necessary, admission for enteral and parenteral nutritional support was a key intervention. All patients received broad-spectrum antibiotics pre-operatively to treat super-imposed infection of MCD lesions. Smokers were engaged in smoking cessation programmes and started nicotine replacement therapy. Steroid treatment was weaned, anaemia corrected with intravenous iron infusions and surgery timed between biologic doses. Pre-operative assessment by a consultant anaesthetist and an individualised plan for peri- and post-operative analgesia was undertaken in each case. All patients were offered psychological support through a clinical psychologist.

### Interventions

After optimisation, all patients underwent surgical procedures tailored to their specific clinical scenarios. Procedures are detailed in Table 2 and described in ‘Results’. Following surgery, patients received specialist post-operative wound care focused on intensive flap monitoring, infection control and nutritional optimisation. They were nursed to avoid pressure on the flap and supported with early mobilisation. Advanced medical therapies were determined on an individual basis by multi-disciplinary discussion.

All patients were followed up regularly in a specialist complex wound clinic attended by consultant plastic and colorectal surgeons and specialist tissue viability nurses. Wound healing status, determined by clinical evaluation, is reported at 6 and 12 months. Wound healing was classed as ‘healed’ (total resolution of cutaneous lesions), partial healing (reduced but ongoing presence of a wound or cutaneous lesion) or no improvement. Minimum follow-up was 12 months with up to 5 years long-term follow-up.

### Statistics

Descriptive statistics are used to summarise patient characteristics and phenotypic traits. Categorical data are presented as frequencies and percentages. Phenotypic trait co-occurrences were visualized using a heatmap illustrating the number of patients who simultaneously exhibited specific pairs of traits. No inferential statistical tests were applied owing to the descriptive nature of the analysis and the relatively small sample size.

## Results

Between January 2019 and January 2024, 15 patients were diagnosed with MCD. Three patients had complete healing of their skin lesions with medical treatment and did not require surgical intervention. One patient had severe MCD with extensive lesions refractory to medical therapy but was too frail for any surgical intervention. Eleven operated patients were included in this series. Eight (72.7%) patients had florid granulomatous inflammation of the skin on histological examination. Three (27.3%) patients had accumulation of multi-nucleate giant cells in the dermis, with an inflammatory lymphocytic infiltrate.

### Patient demographics

All 11 patients were White: English, Welsh, Scottish, Northern Irish or British females. Median age at time of surgery was 37 years, ranging from 20 to 74 years. Median body mass index was 24.4 kg/m^2^, ranging from 20.4 to 40 kg/m^2^ (Table 1). One patient was an active smoker, with the remaining ten being non- or ex-smokers. One patient was an insulin-dependent diabetic. Smoking cessation and optimisation of glycaemic control were undertaken pre-operatively.

**Table 1:** Patient demographics and treatment history

Case	Demographics (age, sex, ethnicity)	Montreal classification	Smoking status	BMI (kg/m^2^)	Previous medical treatment	Previous surgery
1	31 years, F, Caucasian	A2 L2 B1p	Non-smoker	20.4	Prednisolone, azathioprine, adalimumab, ustekinumab, vedolizumab, 0.1% prolonged courses of antibiotics	Defunctioning ileostomy (2019)
2	37 years, F, Caucasian	A2 L2 B1p	Non-smoker	32.5	Prednisolone, azathioprine, mercaptopurine, methotrexate, infliximab, adalimumab, ustekinumab, vedolizumab, 0.1%, prolonged courses of antibiotics	Subtotal colectomy and end ileostomy (2009)
3	74 years, F, Caucasian	A3 L2 B1	Ex-smoker	24.4	Prednisolone, azathioprine, infliximab, ustekinumab, prolonged courses of antibiotics	Defunctioning ileostomy (2019)
4	26 years, F, Caucasian	A1 L2 B1p	Non-smoker	22.8	Prednisolone, azathioprine, mercaptopurine, adalimumab, prolonged courses of antibiotics	Defunctioning ileostomy (2012)
5	56 years, F, Caucasian	A2 L2 B1p	Ex-smoker	24.3	Prednisolone, azathioprine, infliximab, vedolizumab, prolonged courses of antibiotics	Subtotal colectomy and end ileostomy (2014)
6	27 years, F, Caucasian	A2 L3 B1p	Non-smoker	20.8	Prednisolone, azathioprine, infliximab, adalimumab, prolonged courses of antibiotics	Ileocaectomy and ileocolostomy (2014)
7	50 years, F, Caucasian	A2 L3 B3p	Ex-smoker	24.2	Prednisolone, azathioprine, methotrexate, infliximab, prolonged courses of antibiotics	Ileocaecectomy and ileocolostomy (2013)
8	37 years, F, Caucasian	A2 L2 B1p	Non-smoker	36.4	Prednisolone, azathioprine, infliximab, adalimumab, ustekinumab, prolonged courses of antibiotics	Defunctioning ileostomy (2019)
9	55 years, F, Caucasian	A2 L2 B3	Ex-smoker	30.1	Prednisolone, azathioprine, infliximab, adalimumab, prolonged courses of antibiotics	Panproctocolectomy and end ileostomy (2006)
10	29 years, F, Caucasian	A2 L2 B3p	Non-smoker	28.9	Prednisolone, azathioprine, infliximab, ustekinumab, prolonged courses of antibiotics	Defunctioning colostomy (2018), panproctocolectomy and end ileostomy (2019)
11	20 years, F, Caucasian	A2 L2 B1	Smoker	40.0	Prednisolone, azathioprine, infliximab, prolonged courses of antibiotics	Defunctioning ileostomy (2022)

### Luminal CD phenotype

**Fig. 1:**
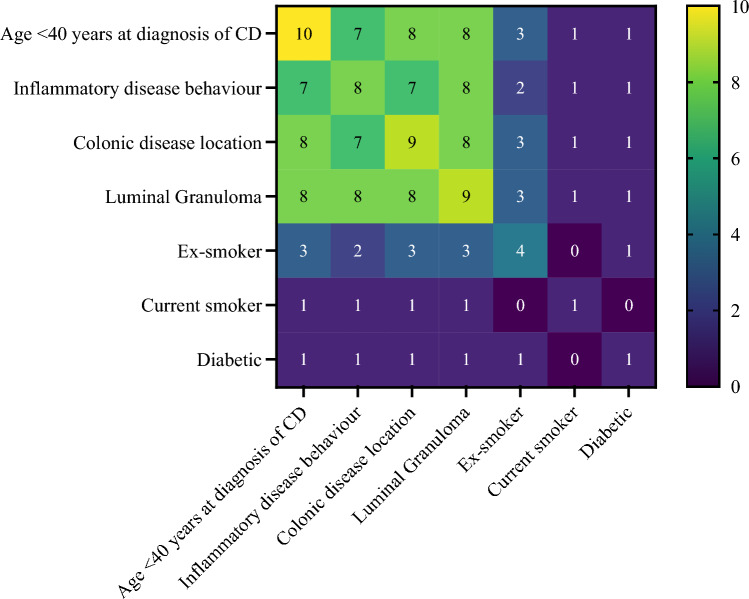
Phenotypic co-occurrence in patients with MCD. Heatmap visualising the co-occurrence of phenotypic traits among patients. Each row and column represent a specific phenotypic trait, and the values indicate the number of patients exhibiting both traits simultaneously. The colour bar on the right provides a reference for interpreting the co-occurrence counts. CD, Crohn’s disease

**Fig. 2:**
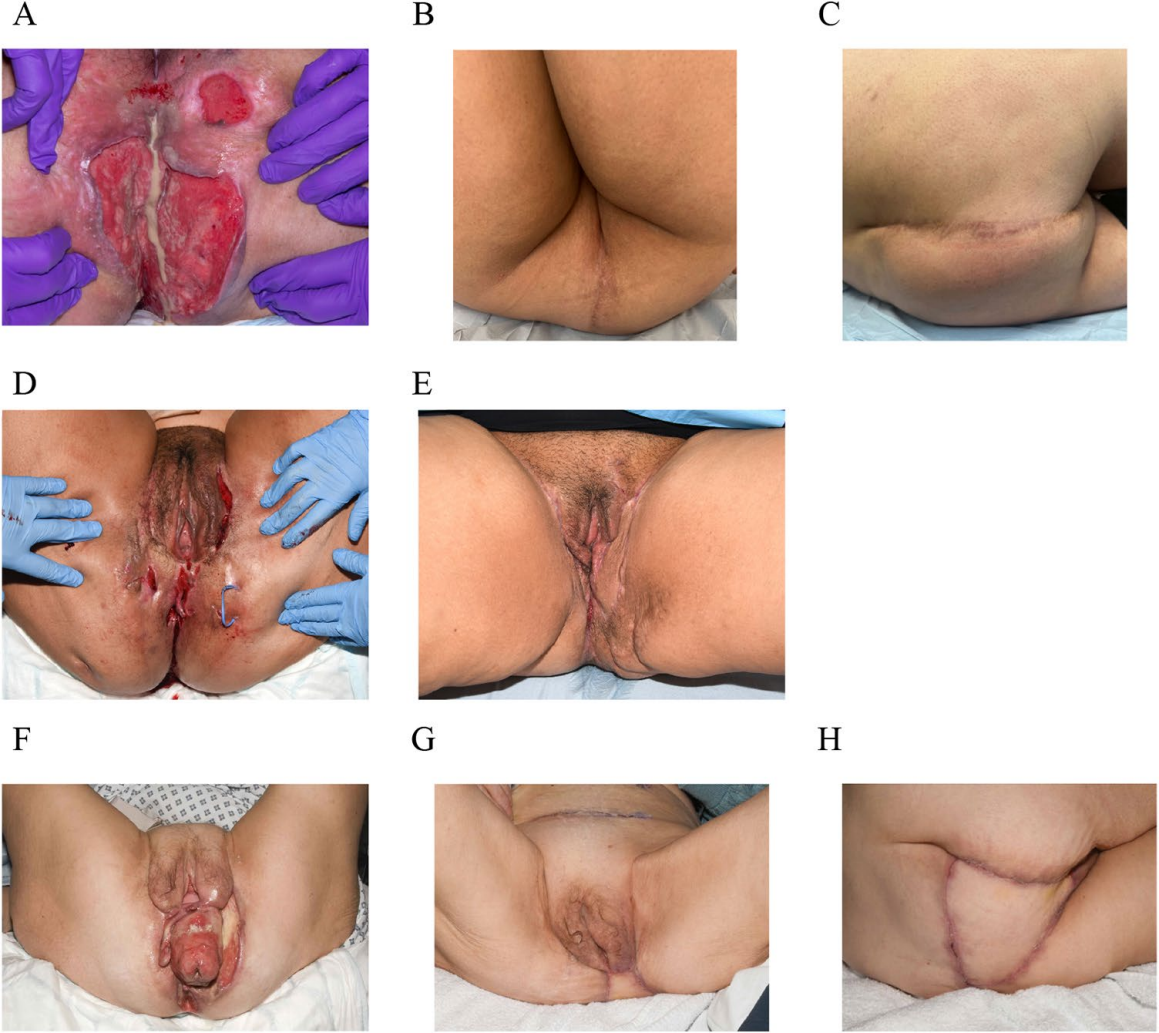
Pre-operative and post-operative clinical images of patients undergoing surgical management of MCD. **A** Case 11: pre-operative left lateral view, **B** post-operative lithotomy and **C** left lateral view. **D** Case 2: pre-operative lithotomy view and **E** post-operative lithotomy view. **F** Case 3: pre-operative lithotomy view, **G** post-operative lithotomy and **H** left lateral view

The median age of luminal CD diagnosis was 27 years, ranging from 16 to 73 years. Ten (90.9%) patients were diagnosed under the age of 40 years. Nine (81.8%) patients had granulomas present on luminal biopsies (Fig. 1). Distribution of luminal CD was colonic in nine (81.8%) cases and ileocolonic in the remaining two. None had luminal CD affecting the upper gastrointestinal tract or isolated ileal disease. Inflammatory disease behaviour was present in eight (72.7%) cases, with the remaining three cases having a penetrating behavioural phenotype. Eight (72.7%) patients had perianal fistulae present on Magnetic Resonance Imaging (MRI). On pre-operative MRI and clinical examination under anaesthesia, the cutaneous lesions were distinct from identified fistula tracks, as demonstrated by the groin lesions seen in Fig. 2D.

### MCD lesions

All patients had ulcerating MCD lesions affecting the perineum (the skin between the anus and external genitalia). The groins (54.6%) and external genitalia (45.5%) were the next most affected sites. In this series, lesions were classed as affecting the genitalia if ulceration involved the labia majora and/or labia minora. Distant lesions in the abdominal wall were present in three cases, specifically in intertriginous areas, and the axilla involved in two cases. Seven (63.6%) patients had multiple affected sites, with five (45.5%) patients having three or more affected areas.

### Previous treatment

Prior to definitive surgical treatment, all patients had received courses of oral corticosteroids, antibiotics, azathioprine and topical tacrolimus ointment—with persistent lesions. Anti-tumour necrosis factor (TNF) therapies had been used in all cases, with seven (63.6%) having two or more biologics and three (27.3%) with three or more biologics. Ustekinumab was used in five (45.5%) patients, and vedolizumab in three (27.3%) patients.

At referral, all patients had already undergone surgery to form a stoma, with four (36.4%) patients having already had a major colonic resection as part of their prior treatment. Two (18.2%) patients had undergone a proctectomy for CD, in their referring centre, and were referred having developed recurrent MCD lesions refractory to medical treatment.

### Operative management

Surgical interventions were individualised to each patient’s specific clinical scenario, performed by consultant colorectal and plastic surgeons and described in Table 2.

**Table 2:** Surgical treatment and wound healing outcomes

Case	Surgical treatment	Perineal reconstruction technique	6-Month healing outcome	12-Month healing outcome	Post-surgical medical treatment	Further surgical treatment	Total length of follow-up (months)	Outcome at final follow-up
1	Laparoscopic panproctocolectomy	V–Y advancement flap	Healed	Healed	None	None	13	Healed
2	Open completion proctocolectomy	Lotus petal flap V–Y advancement flap	Partial healing	Partial healing	Tacrolimus	Excision of non-healing ulcer left mons pubis	32	Healed
3	Laparoscopic panproctocolectomy	V–Y advancement flap	Healed	Healed	None	None	57	Healed
4	Laparoscopic panproctocolectomy	N/A	Partial healing	Partial healing	Tacrolimus	Healed at last follow-up	62	Healed
5	Open completion proctocolectomy and excision of abdominal wall	V–Y advancement flap	Partial healing	Partial healing	Tacrolimus ustekinumab	Re-excision of abdominal wall lesion	48	Partial healing
6	Laparoscopic proctocolectomy	Gluteal flap	Partial healing	Partial healing	Tacrolimus HBOT	Re-excision of natal cleft ulcer and V–Y advancement flap	51	Healed
7	Open completion proctocolectomy	N/A	Healed	Healed	None	None	15	Healed
8	Open panproctocolectomy and perineal reconstruction (gluteal flap)	Gluteal flap	Healed	Healed	None	None	37	Healed
9	Transperineal excision of perineal sinus and perineal reconstruction (gluteal flap)	Gluteal flap	Partial healing	Partial healing	Tacrolimus adalimumab	Re-excision of non-healing ulcer	36	Healed
10	Excision of retained mesorectum, perineal sinus and peritoneal inclusion cyst and perineal reconstruction (gluteal flap)	Gluteal flap	Partial healing	Partial healing	Tacrolimus HBOT ustekinumab upadacitinib	None	19	Partial healing
11	Laparoscopic panproctocolectomy	N/A	Healed	Healed	Adalimumab	None	12	Healed

Nine (81.8%) patients underwent proctocolectomy or completion proctectomy at our centre. Proctectomy was performed with a total mesorectal excision and intersphincteric dissection, with a laparoscopic approach used in five (55.6%) patients. Six (66.7%) patients underwent perineal skin and subcutaneous tissue reconstruction at the time of their proctectomy/proctocolectomy. Perineal reconstruction was undertaken using a V–Y advancement flap for three patients (case 1, 3 and 5). A gluteal flap was used in two patients (case 6 and 8), with one patient (case 2) requiring a combination of a lotus petal and advancement flap to achieve adequate reconstruction (Fig. 2). Abdominal flaps were not used to avoid further abdominal wall disruption in a cohort of patients who had already undergone multiple abdominal surgeries and had a high risk of abdominal incisional hernia. The remaining three patients had primary closure of their perineal defect by the plastic surgery team. All patients were managed in a high-dependency unit in the immediate post-operative period.

Two (18.2%) patients (case 9 and 10) had already undergone a panproctocolectomy for their CD and subsequently referred with symptomatic, non-healing perineal ulcers clinically and histologically consistent with MCD after biopsy of the skin. MRI scans of the perineum were performed to define the anatomy of the post-proctectomy sinus. For case 10, the original proctectomy was performed with a close-rectal dissection, so an abdominal-pelvic approach was undertaken to excise the remaining mesorectum, along with the ulcerated perineal skin and the perineum reconstructed utilising a gluteal flap. Granulomatous inflammation of the skin was found on histology and histological examination of the remnant mesorectum found persistent granulomatous inflammation—a potential driver of cutaneous recurrence.

### Wound healing outcomes

Following surgery and inpatient discharge, patients were reviewed monthly in a complex wound clinic and received specialist care from experienced tissue viability nurses. In addition to regular follow-up by colorectal surgery, specialist IBD gastroenterologists, dieticians, specialist pharmacists and IBD nurse specialists supported their recovery.

Healing outcomes were assessed clinically at 6 and 12 months post-surgery. At 6 months, five (45.5%) patients achieved complete wound healing, while six (54.5%) had persistent unhealed wounds, albeit with a lower overall wound burden. This remained the same at 12 months. However, at long-term follow-up (median 36 months), nine (81.8%) patients had complete wound healing. No patients experienced a recurrence of ulceration after complete healing at the time of follow-up. Surgery made a positive clinical impact on all the patients in this series.

### Post-operative treatment

The six patients with unhealed wounds at 6 months went on to receive additional treatment. This was given in an escalated manner, with topical 0.1% tacrolimus, oral antibiotics, escalation of advanced medical therapies and Hyper-Baric Oxygen Treatment (HBOT). Four patients went on to have surgical local re-excision of small non-healing areas after biopsies demonstrated persistent granulomatous inflammation, achieving complete wound healing in three (75%) cases.

## Discussion

This study highlights the complexities of managing MCD, a rare and challenging extra-intestinal manifestation of Crohn’s disease. Our findings emphasise the critical role of a combined medical and surgical multidisciplinary approach, and the need for individualised surgical treatment strategies to improve outcomes for patients with this debilitating condition.

Specifically, we showed that rigorous phenotyping and tailored surgical intervention combined with medical therapy, thorough pre-operative optimisation, close MDT follow-up and wound care resulted in long-term improvements in wound healing outcomes; 45.5% of patients achieved complete healing by 6 months, with a healing rate that increased to 81.8% at final follow-up (median 36 months). We stress the importance of pre-operative optimisation to address modifiable risk factors such as active steroid use, anaemia, malnutrition (weight loss > 10%), smoking and poorly controlled diabetes in achieving these results. If pre-operative optimisation is not possible, a staged operative strategy should be considered with a delayed proctectomy.

This study emphasises the recurrent and refractory nature of MCD. All patients had a severe disease phenotype requiring multiple treatment modalities, with symptoms persisting despite the use of multiple biologic agents and faecal diversion. Nine (81.8%) patients had granulomas evident on luminal biopsy histopathology, another marker of an aggressive disease phenotype [6].

An important observation from our cohort is the distinction between MCD and pCD. Although these phenotypic entities frequently coexist and overlap, our findings support the concept that MCD represents a separate cutaneous manifestation of CD rather than an advancement of refractory perianal disease. Of the 11 patients undergoing surgery for MCD, 8 had perianal fistulae at the time of operation, 3 patients had no fistulising pCD and all 11 patients had histological evidence of non-caseating granulomatous inflammation within the skin. This supports the hypothesis that MCD can occur alongside or independently of fistulising pCD and should not be regarded solely as a complication or progression of it, warranting separate diagnostic and therapeutic consideration [7].

Currently there are no accepted guidelines for the management of MCD. Biologics targeting TNF-alpha, interleukin (IL)-12/23 and IL-17 inflammatory pathways have a well-established role in treating immune-mediated inflammatory skin diseases such as psoriasis and hidradenitis suppurativa [8, 9]. However, their efficacy and use for cutaneous manifestations of CD is poorly understood. A ‘top-down’ treatment approach for luminal CD, with early initiation of biologics, was shown to be highly effective and safe and result in sustained steroid and surgery-free remission in the recent PROFILE trial [10]. Emerging evidence suggests a potential role for Janus kinase (JAK) inhibitors in the management of refractory MCD. A recent report in *JAMA Dermatology* described successful treatment of metastatic Crohn’s disease with JAK inhibition, highlighting its efficacy in inflammation resistant to conventional therapies [11]. In addition, experience from the UK has demonstrated benefit of JAK inhibitors in the treatment of chronic post-proctectomy perineal sinus in CD [12]. Although data remain limited, these observations support further exploration of JAK inhibition as a therapeutic option in selected patients with refractory disease.

Very little has previously been published on the role of surgery in the management of MCD. The largest series to date was in 1993, in which Williams et al. reported surgical debridement of post proctectomy skin lesions in five patients [4]. The paucity of evidence to guide the surgical approach in those requiring proctectomy for CD is an area of unmet clinical need, with unhealed perineal wounds and persistent perineal symptoms seen in up to 45% of patients [13–15]. The role of total mesorectal excision (TME) at time of proctectomy is debated, but the concept of the mesentery being an immunological driver of disease recurrence in CD is gaining traction [16–19]. TME is the approach undertaken for patients undergoing proctectomy in our institution, as close-rectal dissection (CRD) has previously been identified as a risk factor for perineal wound complications [19]. This is cautiously supported by our finding of persistent granulomatous inflammation in the retained mesorectum of one patient who had previously had proctectomy with CRD and developed MCD in the perineal wound.

Primary skin closure may be suitable for small, superficial ulcers, but is often not an option in severe cases owing to the poor quality of inflamed tissue, poor wound healing, significant soft tissue destruction and high risk of breakdown [20]. Excision of the cutaneous ulceration with subcutaneous flap-based reconstruction after inter-sphincteric proctectomy is needed when soft tissue destruction compromises function, particularly in anovaginal ulceration, posterior vaginal wall defects and perineal body loss, commonly seen in these cases. The authors favour excision of the cutaneous ulceration if possible, to reduce the wound burden and improve quality of life, although the lack of quality-of-life data is a limitation of this study. Ulceration of the external genitalia is not excised to preserve tissue and reduce functional complications and scaring.

Common reconstructive techniques include the V–Y advancement flap, which allows local tissue preservation while mobilising healthy skin and subcutaneous tissue to cover perianal defects. Gluteal flaps are better suited for larger perineal cutaneous wounds requiring bulk tissue replacement [21]. The lotus petal flap, a perforator-based fasciocutaneous flap harvested from the gluteal region, provides well-vascularised, sensate soft tissue, making it an excellent option for perineal and vaginal wall reconstruction. The choice of reconstructive flap must be tailored to each patient, with the aim of achieving structural support, functional restoration and good cosmetic outcomes with minimal donor site morbidity [22].

In our series, re-excision of residual lesions or sinus tracts was necessary in some cases, reflecting the complex and aggressive disease course. Clinicians should continue medical therapy as risk of recurrence is high. Combined medical and surgical management along with adjuncts to wound management such as topical 0.9% tacrolimus, HBOT and re-excision should all be considered in the management of recurrent lesions. We report use of HBOT in two of our patients, resulting in a complete clinical response in one patient. There is some evidence supporting the use of HBOT in the treatment of severe post-proctectomy wound complications and recurrent perineal sinus [23–26].

This study is limited by the retrospective nature of its design and lack of a comparator group of CD proctectomy without MCD involvement. However, given the rarity of this condition, and lack of previously published data on outcomes in those requiring surgery for MCD, this study represents the largest national cohort of patients undergoing surgical management for MCD and provides an early assessment of the clinical features, surgical management and wound healing outcomes for this aggressive disease.

## Data Availability

No datasets were generated or analysed during the current study.
